# Regional Private Financing Risk Index Model Based on Private Financing Big Data

**DOI:** 10.3389/fpsyg.2022.874412

**Published:** 2022-04-11

**Authors:** Jingfeng Zhao, Bo Li

**Affiliations:** College of Management and Economic, North China University of Water Resources and Electric Power, Zhengzhou, China

**Keywords:** private financing, financing risk, principal component analysis, private lending, market's effectiveness

## Abstract

With the rapid development of China's economy in recent decades, and the decentralization of the country's economic regulation and legal support, private financing has developed rapidly due to its simple, flexible and unique advantages. Some SMEs can solve it to some extent through private financing. The company's own financing issues have also helped the local financial market's effectiveness. Based on the “Yantai Private Financing Interest Rate Index,” this paper constructs a private financial risk index model from three perspectives of interest rate risk, scale risk and credit risk, and conducts a case simulation analysis of the private financing risk index. The characteristic indicators of the early warning system are screened from the macro, micro and stability dimensions, and subjective and objective adjustment coefficients are set for each indicator from both subjective and objective perspectives. This article takes the Yantai Index as the representative of China's private financing interest rate index. Based on the term structure of Yantai's private lending rate, this paper studies its response to macroeconomic shocks and analyzes the information value it contains. And use the private financing interest rate index to build a financial risk monitoring model. Through the system transformation model, the article finds that there is a significant asymmetry in the response of private lending to macroeconomic shocks. When private lending rates are higher, inflation has a greater effect on interest rates; when private lending rates are lower, monetary policy has a stronger regulatory effect on private lending rates. In the data processing, the principal component analysis method and the Bayesian vector autoregressive model were established. Through the study of this article, it is concluded that the interest rate decreases with the increase of the term, and the risk comparison is performed for the 1-month period, 3-month period, June period, 1-year period, and more than 1-year. The risks in the previous period are greater, and the risks in the March and June periods are relatively small. This model can be used to calculate the comprehensive evaluation value and its fluctuation in the historical risk market and historical equilibrium market, so as to determine the risk range of the comprehensive evaluation value. Thus, the early warning system is verified to be feasible.

## Introduction

Private finance refers to all financial transaction activities for the purpose of profit that are separated from the financial institutions established by the competent authorities of the state in accordance with the law. As an integral part of the financial system, private finance has a long history of development, plays an indispensable role in economic development, and supplements formal finance to a certain extent. Private finance is conducive to the development of the private economy. Although the private economy plays an important role in the national economy, it has been constrained by the financial industry for a long time, its development scale has been restricted to a certain extent, and the financing of SMEs has often been hindered. To some extent, financing difficulties have become a common problem for SMEs. Banks and other financial institutions tend to invest more credit funds in state-controlled large and medium-sized enterprises (Yu et al., 2015). In the process of lending to banks, SMEs are very cumbersome and often encounter difficulties. With the rapid development of the private economy, it needs a lot of credit funds to support it. However, it is difficult for the private economy to obtain the required funds through formal financial channels such as bank loans. In this case, private financing emerged. Private loan income is high (Murthy and Blackstone, 2015; Chen et al., 2017). In the early years, China's regional financial operation report pointed out that after the country strengthened macroeconomic control, due to the limited amount of direct financing available to enterprises, SMEs in economically developed areas tend to look for other financing channels, such as private loans, related financing, and lease financing. In this case, interest rates on private lending have increased significantly. The private loan market plays an indelible role in alleviating the shortage of credit funds (Yang, 2015). On the other hand, despite the continuous growth of Chinese residents' income and per capita disposable income, Party C still has limited formal investment models, mainly through real estate, savings deposits, government bonds, stocks, bonds, funds, futures, options and other financial Derivatives (Liang, 2016). However, the rate of return on savings deposit products is relatively low, and due to the impact of inflation expectations, currency deposits may bear the risk of depreciation after depositing in the bank. At the same time, at present, the types and issuance of Chinese government bonds cannot meet the investment needs of residents (Zhang et al., 2015). Products such as stocks, funds, bonds, futures, and options have high investment risks or high investment thresholds, and require professional knowledge reserves, which is not conducive to the active participation of residents. The fourth type of fixed asset investment has higher capital thresholds for investors, longer investment periods, lower returns, poor liquidity, and low enthusiasm for residents' participation. On the contrary, residents are more willing to invest in private lending markets where interest rates are higher than bank deposit rates, with risks between savings and stocks, and lower thresholds (Shang et al., 2021; Yin et al., 2022). At present, due to various reasons such as imperfect credit reporting system, information asymmetry, and the market is not fully regulated, all regions are faced with a strong demand for a sunny, standardized and healthy private financing market. Although local finance has issued many policies for private supervision, and the supervision has been continuously strengthened, there is still a lack of a feasible private financial early warning and monitoring system.

Formal finance requires strict credit assessment and risk assessment of companies applying for loans. The borrowing threshold is high, the approval procedures are cumbersome, and the approval cycle is long. Therefore, its main service target is large and medium-sized enterprises that have already performed credit ratings. And private finance has obvious information advantages, cost advantages, and geographical advantages. Therefore, in the financial market, private finance is more easily accepted by small and micro enterprises than formal finance (Ma and Lv, 2019; Liu, 2022). At the same time, since private lending generally occurs in the region, and both borrowers and lenders are familiar with each other and have a deeper understanding of each other, before the loan takes place, the company's credit situation can be basically grasped without the need for a complicated corporate survey, and the borrowing cost is low; After the borrowing occurs, the lender can track and supervise the operating status, profitability, and repayment ability of the borrowing enterprise in order to recover the loan in a timely manner and control the borrower's default risk. Information advantages, geographical advantages, and cost advantages make private financing as a supplement to formal finance, and it is easier to be favored by small and micro enterprises. It has unparalleled advantages in small and micro enterprises financing. Private finance is more closely matched with the behavior of small and micro enterprises. Similar to the loose operation and management of small and micro enterprises, the funds of private finance mostly come from idle funds in the individual and private capital of urban and rural residents, and there is a strong freedom in management, which makes private finance and small and micro enterprises have behavioral models. Both pursue simpler loan procedures. At the same time, due to the lack of effective risk assessment methods for private lending, in order to reduce the risk of investment or lending, private lending behaviors often occur in the region, among acquaintances, or through acquaintances (Wang et al., 2021; Xu et al., 2022). This method based on regional characteristics or through the use of acquaintances is convenient for lenders to understand and track the borrower. This kind of restriction of civil financing behavior in regional circles and acquaintance circles relies on “morality” and long-term games, and establishes a set of restraining means different from the formal financial law punishment. The frequency, small amount, and urgency of financing of small and micro enterprises are exactly matched with the behavioral characteristics of private finance, which promote mutual development. Private finance has played a huge role in optimizing resource allocation and increasing capital utilization. In the course of long-term economic development, a large amount of capital has gradually accumulated. As mentioned earlier in this article, small and medium-sized enterprises require a large amount of credit funds as a support during the development process to provide an output path for the accumulated capital (Yoo and Choi, 2015; Zhou et al., 2017). In addition, private finance has information, geographical, and cost advantages, to a certain extent, guarantees that private finance can more accurately determine the credit risk, asset status, and solvency of borrowing objects, so it can make potential risks for borrowing behavior. More accurate trade-offs. At the same time, it can transform saving behavior into investment activities, provide export channels for the remaining idle funds in economic activities, optimize social resource allocation, and improve capital utilization efficiency.

P. Li's research group proposed the use of vector autoregressive model (VAR) analysis to explore the establishment of a measurement and early warning system for private financial risks to provide decision-making references for the prevention and resolution of regional private financial risks (Singh et al., 2021). Cao proposed a method to innovate the risk semaphore level, and effectively combined it with the time series ARMA model and gray prediction theory, which can not only accurately assess the risk level of the past few years, but also continuously predict future financial risks (Chen et al., 2018; Ko and Marreiros, 2021). The FAN Wei-long research group proposed a financial risk early warning model based on Bayesian neural network, and conducted empirical tests using economic and financial data from 1993 to 2017. The results show that the Bayesian neural network financial risk early-warning model describes the uncertainty of parameter estimation and risk early-warning through posterior distribution and prediction distribution, and introduces zero-mean normal distribution to enrich the risk early-warning information. In the case of prior distribution, the problem of overfitting for small data set modeling is avoided (Li and Zhang, 2017). Zheng suggested establishing a financial risk early-warning indicator system that includes Internet financial risk perception, and analyzed how to use web crawler technology to collect and process data so that each indicator is comparable in the same system. On this basis, according to the characteristics of high-dimensional non-normal data, a RAGA projection tracking model was constructed, and the non-parametric method was used to find the projection variables that could best reflect the characteristics of multi-dimensional data and projected. Multi-dimensional observation data enters the low-dimensional subspace to better realize early warning of financial risks (Cao et al., 2016; Hong et al., 2019). Over-sampling technology (SMOTE) is used to improve the over-fitting of the support vector machine (SVM) unbalanced sample learning ability, and the adaptive synthesis sampling method (ADASYN) and the optimization of reducing down-sampling are introduced. ODR sampling overcomes the blindness of smoking and the limitation of processing objects when generating new samples, and then combines with SVM to build an improved SVM, namely, the ODR-ADASYN-SVM model to predict extreme financial risks in China. Finally, a *t*-test is used to test the difference in prediction accuracy of each model and to evaluate the prediction stability of each model. Obviously, the ODR-ADASYN-SVM model can not only significantly improve the learning ability of SVM imbalance samples, but also effectively overcome the overfitting of oil fume, thus showing the excellent performance of extreme financial risk prediction (Zheng et al., 2015; Fan and Wang, 2016).

This article takes the Yantai Index as the representative of China's private financing interest rate index, and based on the term structure of Yantai's private lending rate, studies its response to macroeconomic shocks and analyzes the information value it contains. And use the private financing interest rate index to build a financial risk monitoring model (Ahmed and Saad, 2019; Mohammed, 2021). Similarly, the model will be established at different interest rates for different maturities, financing rates for different financing products, and different loans in the Yantai region Based on the data of the entity's loan transaction volume, non-governmental financial risk is monitored through three aspects: interest rate risk, scale risk and credit risk. After constructing a theoretical model, a period of Yantai index is substituted into the model for example analysis (Zhang et al., 2018). The entire early warning system is mainly based on the selection of grass-roots data, which requires a large amount of reliable and real data to support, and the amount of data will directly affect the validity of the entire model. Therefore, ensuring the source of data selection is to ensure the validity of the model. In addition, the division of indicators and the establishment of models are flexible, and can be improved in the later application process as the actual operation needs to be improved. Adaptive changes can be made with the needs of the market to ensure immediate effectiveness of the entire system. The operation of the whole system is close to the reality, which can make the monitored specific data more effectively reflect the abstract state of the market, and help to promote the healthy operation of the market economy. Furthermore, by analyzing the similarities and differences of the comprehensive indicators in the market equilibrium state and the market risk state, the risk area is divided according to the proximity of the market comprehensive assessment value and the two types of market state values, which is used to judge the risk state of the monitoring market. In the same way, according to the numerical difference of each indicator in the two types of markets, the risk status of each early warning indicator can be divided, and the corresponding monitoring status area under different values of the indicator can be defined, so as to determine the essential influencing factors of the market risk. Provide theoretical support for taking targeted measures.

## Proposed Method

### Principal Component Analysis

The basic principle of principal component analysis is based on a mathematical transformation that extracts a certain number of unrelated complex variables from many interconnected basic variables. The extracted composite variables retain the information characteristics of the basic variables, so we can study the original basic variables by analyzing the new composite variables. The newly extracted composite variable is called the main component of the original variable. This method can effectively reduce the amount of data, and is a statistical method for studying and testing the variance or covariance structure of a compound variable factor system.

In the study of the term structure of private lending rates, this article uses a continuous function of terms to represent the spot interest rates of different terms. Therefore, select a certain number of discrete loan terms from the current interest rate term. The points represented by these discrete terms on the corresponding yield curve are called backbone points, and different backbone points represent different levels of backbone interest rates. Changes in interest rates are the basic variables mentioned above.

The principal component can be expressed as a linear combination of changes in the main interest rate:


(1) $$\begin{array}{l} p_{i}=\overset{n}{\underset{j-1}{\sum }}p_{i,j}\Delta r_{j} \end{array}$$

*p*_*i*_ is the main component, Δ*r*_*j*_ is the main interest rate change value, and *p*_*i,j*_ is the main component coefficient. The matrix form of formula (1) is:


(2) $$\begin{array}{l} \left[\begin{array}{c} p_{1}\\ ...\\ p_{n} \end{array}\right]=\left[\begin{array}{ccc} p_{1,1} & ... & p_{1,n}\\ ... & ... & ...\\ p_{n.1} & ... & p_{n,n} \end{array}\right]\times \left[\begin{array}{c} \Delta r_{1}\\ ...\\ \Delta r_{n} \end{array}\right] \end{array}$$

[*p*_*i,j*_] is the main component coefficient matrix, [*p*_*i*_]and[Δ*r*_*j*_]are the main components and vectors of the main interest rate changes. The principal component coefficient matrix is obtained from the transaction fund price data of the private financing market in Yantai. By selecting the corresponding number of major interest rates, in this model, the data selected are the personal loan interest rates for January, March, June, 1 year and more. In a certain period of time, according to the corresponding specific interval, observe the specific amount of interest rates in different financing cycles, and then perform a first-order differential processing on the observed value of the interest rate level to obtain the interest rate. Finally, change the data sequence to obtain the corresponding The main interest rate of the interest rate level of the financing cycle, and a matrix W is formed according to the change of the main interest rate. By calculating the covariance matrix of the matrix, it is verified whether the matrix e formed by the eigenvectors is the coefficient matrix of the main component. The verification method is shown in formula (2):


(3) $$\begin{array}{l} \sum \left[\begin{array}{c} p_{i,1}\\ ...\\ p_{i,n} \end{array}\right]=\lambda_{i}\times \left[\begin{array}{c} p_{i,1}\\ ...\\ p_{i,n} \end{array}\right] \end{array}$$

The specific objectives of the principal component analysis method are divided into the following three categories: study the specific changes in each major interest rate level, especially the relationship between the volatility of interest rate levels and the length of the loan repayment period; study the correlation between the interest rates of different major components The direction and quantity of the correlation between changes in loan interest rates in different periods; determine the effective principal component ratio to describe the change in interest rate terms, and determine the degree of interpretation of the principal component on the interest rate curve.

The overall volatility on the interest rate curve can be used to measure the overall volatility of all major interest rates on the curve. In the data, it is the sum of the λ_*i*_obtained, i.e., the total volatility $\overset{n}{\underset{j=1}{\sum }}\lambda_{j}$. The main component's ability to explain overall volatility is the proportion of this component that explains overall volatility, which can be expressed as $\varepsilon_{i}=\lambda_{i}/\overset{n}{\underset{j=1}{\sum }}\lambda_{j}$. The explained proportion of the principal component provides a link between interest rates in different periods of the interest rate curve, so it can use the explained proportion data of the first few principal components to determine the necessary number of principal components to describe the change in interest rates. Interest rate curve.

### Basic Orinciples of Bayesian Vector Autoregressive Models

The vector autoregressive model is a single time series model. It selects a variable with a certain correlation to form a vector system. The correlation between the variables in the system is expressed by multilevel lag regression of the variables. However, when a large number of data samples are needed to estimate the parameters of this non-limiting model, when the data series is short, the accuracy of the estimation results of the VAR model will be affected to some extent. The Bayesian inference method proposed an effective solution to this problem. The VAR model that uses Bayesian methods to estimate parameters is the BVAR model. The model uses a prior distribution method, which is conducive to better analysis of information on private lending rates. value. The construction of risk monitoring model not only selects reasonable indicators as the key influencing factors of risk monitoring, but also needs to ensure the effectiveness of the entire process, from the classification of indicators, the setting of weights to the establishment of comprehensive evaluation models and the substantial impact on risks. The following principles must be fully considered to ensure the rationality of the whole process.

### Yantai Private Financing Interest Rate Risk Index

This part is composed of the risk interest rate index of the main body of Yantai private financing loan and the risk interest rate index of the term of Yantai private financing loan. Private financing risk level monitoring is the process of screening, measuring, assembling and evaluating a series of indicators reflecting the regional private financing risk level. Considering the role and influence of various factors on financing risk, the entire index system design of constructing the private financing risk monitoring model can be designed as a three-tier framework structure. Private finance has the characteristics of high loan interest rate, large scale change and high credit risk, which just highlights the vulnerable link of the private financial chain and is also the gathering point of risk occurrence.

#### Compilation of the Yantai Private Financing Loan Subject's Interest Rate Risk Index

Based on the interest rate of each subject during the observation period, calculate the average interest rate of each subject. The name of each topic and the corresponding sample mean representation characters correspond to the following: microfinance companies (*r*_1_), private capital management companies (*r*_2_), social direct loans (*r*_3_), private loan service centers (*r*_4_), rural mutual aid associations (*r*_5_), As well as other market topics (*r*_6_).

First, calculate the average monthly loan interest rate of the six entities in the sample, record the six entities as*r*_*t*1_, *r*_*t*2_, *r*_*t*3_, *r*_*t*4_, *r*_*t*5_, *r*_*t*6_, then compare them with the average of the total sample, and then convert the interest rate of each entity into the loan interest rate risk index LRRI (Loan Interest Rate Risk Index), recorded as*LRRI*_1_, *LRRI*_2_, *LRRI*_3_, *LRRI*_4_, *LRRI*_5_, *LRRI*_6_. Among them, ${LRRI}_{n}=r_{t_{n}}/{\overset{-}{r}}_{n}$. The main interest rate risk index indicates the degree to which each main interest rate deviates from the average of the total number of samples: the larger the positive deviation value, the higher the risk premium of each main interest rate, the larger the market loan. The greater the risk.

Then, the standard deviations corresponding to the six subjects were calculated and recorded as*SDL*_1_, *SDL*_2_, *SDL*_3_, *SDL*_4_, , *SDL*_6_. This will provide a numerical basis for the risk range of loan interest rates for the six types of objects. Based on the average plus standard deviation multiples as the upper bound, the risk level of the loan interest rate of each object can be divided into the following four ranges: lower risk range: *LRRI*_*n*_ ≤ 1; higher risk range: 1 < *LRRI*_*n*_ ≤ 1 + *SDL*_*n*_; high risk range: 1 + *SDL*_*n*_ < *LRRI*_*n*_ ≤ 1 + 2 × *SDL*_*n*_; extreme High-risk range: *LRRI*_*n*_ > 1 + 2 × *SDL*_*n*_. The above risk intervals are mainly used to find relevant indicators of abnormal fluctuations while monitoring risks. In fact, the larger the sample size, the more objective its intuitive value. Therefore, we choose to use the proportion of the sample size of each sub-account in the total sample as the corresponding weight. If y is the total sample size, the corresponding sub-entity sample size is *y*_1_, *y*_2_, *y*_3_, *y*_4_, *y*_5_, *y*_6_, then $Y=\overset{6}{\underset{n=1}{\sum }}y_{n}$; then the risk weight of each entity is ω_*n*_ = *y*_*n*_/*Y*; therefore, the final value of the entity's interest rate loan risk indicator is:


(4) $$\begin{array}{l} LRRI=\overset{6}{\underset{n=1}{\sum }}\omega_{n}\times {LRRI}_{n} \end{array}$$

#### Yantai Private Financing Term Interest Rate Risk Index

The occurrence of private financing risks does not happen overnight, but has a gradual change process. The appearance of financial crises often has the characteristics of suddenness, but the uncertainty and concealment are related to the changes of relevant indicators in the financing market. It is preceded by abnormal changes in some indicators. Therefore, if the relevant indicators can be screened, the sensitive factors with strong comprehensiveness and high correlation can be found, and a practical early warning system can be established in combination with their operability, which can effectively predict the financial risks in the private financing market.

As mentioned above, we calculated the average loan interest rate for all terms in the sample. The term and its average representation characters are as follows: January period (${\overset{-}{r}}_{a1}$), March period (${\overset{-}{r}}_{a2}$), June period (${\overset{-}{r}}_{a3}$), 1 year period (${\overset{-}{r}}_{a4}$) and more than 1 year period (${\overset{-}{r}}_{a5}$). Then calculate the average term interest rate ${\overset{-}{r}}_{at_{n}}$ (n = 1, 2, 3, 4, 5) for each month in the observation period (where n represents the nth term), compare the average term interest rates, convert the term interest rates into term interest rate risk indicators *TRRI*, and record them like *TRRI*_1_, *TRRI*_2_, *TRRI*_3_, *TRRI*_4_, *TRRI*_5_, where ${TRRI}_{n}={\overset{-}{r}}_{at_{n}}/{\overset{-}{r}}_{an}\left(n=1,2,3,4,5\right)$.

Here, the term interest rate risk index indicates the degree of interest rate deviation from the total sample average under different conditions: the greater the positive deviation, the higher the risk of each term interest rate. After calculating the interest rate risk index for all months in the sample, the standard deviations for the five periods are calculated as follows:*SDT*_1_, *SDT*_2_, *SDT*_3_, *SDT*_4_, *SDT*_5_, *SDT*_6_. Similarly, with the change of the final impact source of the observation data, the term interest rate risk of each entity is divided into the following four intervals based on the average plus the standard deviation multiple: the lower risk range: *LRRI*_*n*_ ≤ 1; the higher risk range:1 < *LRRI*_*n*_ ≤ 1 + *SDL*_*n*_; High risk area: 1 + *SDL*_*n*_ < *LRRI*_*n*_ ≤ 1 + 2 × *SDL*_*n*_; very high risk area: *LRRI*_*n*_ > 1 + 2 × *SDL*_*n*_. The proportion of the sample in each period in the total sample is selected as the weight of the overall impact of interest rate risk in different periods. In this way, the interest rate risk weight of each period in the total sample is full of ω_*an*_ = *q*_*n*_/*Y*. Therefore, the final value of the interest rate loan risk index for the period is:


(5) $$\begin{array}{l} LRRI=\overset{5}{\underset{n=1}{\sum }}\omega_{n}\times {TRRI}_{n} \end{array}$$

#### Interest Rate Risk Index of Private Financing and Lending in Yantai

Through the above calculations, we can obtain the values of *LRRI* and *TRRI* for each month. Considering that the weights of both are 1/2, the formula for calculating the *RRI* interest rate risk index of the Yantai Citizens Lending Rate Index is:


(6) $$\begin{array}{l} RRI=\left(LRRI+TRRI\right)/2 \end{array}$$

In summary, the index value of the monthly interest rate risk is calculated.

### Yantai Private Financing Scale Risk Index

This part is also composed of Yantai Private Financing Scale Risk Intensity Index and Yantai Private Financing Scale Risk Width Index. These two parts are summarized and weighted, and then the monthly private financing scale risk index is obtained.

#### Yantai Private Financing Scale Risk Intensity Index

For the strength indicator, we use floating amount for calculation. In all samples, we calculate the total number of samples SM in each month in the form and find the minimum sample size*sm*for each month in the sample coverage period. Compare the floating ratio of monthly *SM* to *sm* with each other, record it as *M*, and *M* = *SM*/*sm*; calculate the value of *M* for each month, and calculate the average $\overset{-}{M}$ of each month, and record it as the current scale risk intensity index $LSI=M/\overset{-}{M}$, calculate The standard deviation of the buoy risk intensity index is recorded as *SDM*; therefore, the risk intensity index can be divided into the following four parts: low risk part: *LSI* ≤ 1: higher risk part: 1 < *LSI* ≤ 1 + *SDM*; high risk part: 1 + *SDM* < *LSI* ≤ 1 + 2 × *SDM*; very high risk part: *LSI* > 1 + 2 × *SDM*;

#### Yantai Private Financing Scale Risk Width Index

For the breadth indicator, we choose the number of loans to reflect. The more loans, the more capital flows, and the larger the scope of influence. We calculate the number of loans per month and record it as *SN*. Similarly, we find the minimum sample size *sn* during the sample coverage period, compare the change in the number of loans *SN* relative to *sn*, record it as *N*, and*N* = *SN*/*sn*; and calculate the average *N* of each month and record it as $\overset{-}{N}$. Similarly, the scale risk width index *LWI*, $LVI=N/\overset{-}{N}$. The standard deviation of the scale risk width index change is recorded as *SDN*. According to the risk width index, it is divided into the following four risk intervals: lower risk interval *LWI* ≤ 1: higher risk interval 1 < *SWI* ≤ 1 + *SDN*: high risk interval 1 + *SDN* < *LWI* ≤ 1 + 2 × *SDN*: extremely high risk interval *LWI* > 1 + 2 × *SDN*:

#### Yantai Private Financing Scale Index

Using the above two indicators, we can calculate the monthly *LSI* and *LWI* values during the sample period. Then, the above two indicators are combined into the indicators of the Yantai Private Financing Scale Index, and the weight of both is set to 1/2. The formula for the size index *SRI* (size risk index) is as follows:


(7) $$\begin{array}{l} SRI=\left(LSI+LWI\right)/2 \end{array}$$

### Credit Risk Index of Yantai Private Financing

#### Yantai Private Financing Credit Risk Default Index

We want to count the amount of money that is due every month but not recovered in time, and record it as ; according to the contract period, the total amount that should be recovered in that month, record as *LM*; the monthly repayment rate can also be expressed as *lm*/*LM*; now assume *LS* = *lm*/*LM*, *LS* represents the proportion of outstanding payments to receivables. Now we need to calculate the average value of the proportion of outstanding loans to repayable debt in each month of the sample as $\overset{-}{L}\overset{-}{S}$. Now the change in this ratio is used to calculate the credit risk default indicator $LBI=LS/\overset{-}{L}\overset{-}{S}$, which represents the degree of change in the proportion of the outstanding amount of each month relative to the average, and further calculates its standard deviation as *SDLM*. The credit risk default indicators can also be divided into four risk intervals: lower risk interval: *LBI* ≤ 1; higher risk interval: 1 < *LBI* ≤ 1 + *SDLM*; high risk interval: 1 + *SDLM* < *LBI* ≤ 1 + 2 × *SDLM*; extremely high risk interval: *LBI* > 1 + 2 × *SDLM*.

#### Yantai Private Financing Credit Risk Debt Extension Index

The degree of debt extension requires us to find out the sample of the previous period of borrowing that has been repaid this month, record its contracted period as *T*, and record the delay time to the actual repayment date as *t*. The extent to which this month's repayment time is extended can be expressed in *t*/*T*. The average value of *LT* = *t*/*T* and the extension of the debt repayment period to the contract term is $\overset{-}{L}\overset{-}{T}$. Now use the degree of extension of the debt repayment time to calculate the credit risk debt extension indicator. Now take the month as the unit, calculate the change of the debt extension rate, let $LTI=LT/\overset{-}{L}\overset{-}{T}$, and calculate its standard deviation, then, similarly, the credit risk debt extension rate index can also be divided into the following four intervals: lower risk interval: *LTI* ≤ 1; High risk range: 1 < *LTI* ≤ 1 + *SDLT*; High risk range: 1 + *SDLT* < *LTI* ≤ 1 + 2 × *SDLT* ; Extreme risk range: *LTI* > 1 + 2 × *SDLT*.

#### Credit Risk Indicators for Private Financing in Yantai

Using the above two indicators, we can calculate the monthly and *LTI* values, and convert the two indicators into Yantai's private financing credit risk CRI (credit risk index) according to the same weight, that is,


(8) $$\begin{array}{l} CRI=\left(LBI+LTI\right)/2 \end{array}$$

### Compilation of Yantai Private Financing Risk Index

Combining the above indicators, the final Yantai private financing risk index is determined by three indicators: interest rate risk, scale risk and credit risk. Considering that the three factors have different degrees of influence on market risk, this paper studies the actual risk samples for many years, observes the changes of the three indicators during the period before the financial crisis, and determines the weight of the three indicators. The type of indicator is determined according to the degree of risk fluctuation of each indicator. Assuming the available risk sample size is *n*, we can use the *x* months before the risk of each sample as the data during the observation period to confirm the changes in these three indicators. For any *e*_*i*_ sample, we need to use the calculation method in the risk monitoring model to calculate the index value of *RRI*, *SRI*, *CRI*, in the months before the risk of the sample, and record them as ${\overset{-}{R}\overset{-}{R}Ī}_{i}$, , *SRI*_*i*_, *CRI*_*i*_, and then find the value in each sample. The average value of these three types of indicators is denoted as ${\overset{-}{R}\overset{-}{R}Ī}_{i}$, ${\overset{-}{S}\overset{-}{R}Ī}_{i}$, ${\overset{-}{C}\overset{-}{R}Ī}_{i}$, and the average value of the three types of indicators in this sample is used to calculate the volatility of the three indicators in this sample. The standard deviation of each factor is used to measure the magnitude of its volatility ratio. Then in the sample *e*_*i*_, the fluctuations of the three in the *x* month are calculated according to the standard deviation formula as follows:


(9) $$\begin{array}{l} {WRRI}_{i}=\sqrt{\overset{x}{\underset{k=1}{\sum }}\frac{1}{x}{\left({RRI}_{ik}-{\overset{-}{R}\overset{-}{R}Ī}_{i}\right)}^{2}} \end{array}$$

(10) $$\begin{array}{l} {WSRI}_{i}=\sqrt{\overset{x}{\underset{k=1}{\sum }}\frac{1}{x}{\left({SRI}_{ik}-{\overset{-}{S}\overset{-}{R}Ī}_{i}\right)}^{2}} \end{array}$$

(11) $$\begin{array}{l} {WCRI}_{i}=\sqrt{\overset{x}{\underset{k=1}{\sum }}\frac{1}{x}{\left({CRI}_{ik}-{\overset{-}{C}\overset{-}{R}Ī}_{i}\right)}^{2}} \end{array}$$

Among them, *RRI*_*ik*_, *SRI*_*ik*_, *CRI*_*ik*_, respectively, represents the value calculated in the *k* month in the *i* sample. After calculating the standard deviation, we can calculate the value of the fluctuations of various factors in the relevant period of the risk according to the ratio of the standard deviation of the sample. The formula is as follows:


(12) $$\begin{array}{l} \alpha =\frac{{WRRI}_{i}}{{WRRI}_{i}+{WSRI}_{i}+{WCRI}_{i}} \end{array}$$

(13) $$\begin{array}{l} \beta =\frac{{WSRI}_{i}}{{WRRI}_{i}+{WSRI}_{i}+{WCRI}_{i}} \end{array}$$

(14) $$\begin{array}{l} \gamma =\frac{{WCRI}_{i}}{{WRRI}_{i}+{WSRI}_{i}+{WCRI}_{i}} \end{array}$$

Among them, *i* represents the *i* risk sample. Therefore, for all *n* samples, we can get different proportions of *n*, calculate the impact value of three types of factors for all samples, and calculate the average as $\bar{\alpha }$, $\bar{\beta }$, $\bar{\gamma }$. As the corresponding weight of these three types of indicators, the following formula is established:


(15) $$\begin{array}{l} \bar{\alpha }=\frac{1}{n}\overset{n}{\underset{i=1}{\sum }}\alpha_{i} \end{array}$$

(16) $$\begin{array}{l} \bar{\beta }=\frac{1}{n}\overset{n}{\underset{i=1}{\sum }}\beta_{i} \end{array}$$

(17) $$\begin{array}{l} \bar{\gamma }=\frac{1}{n}\overset{n}{\underset{i=1}{\sum }}\gamma_{i} \end{array}$$

Considering that the final risk index is represented by RI, the final evaluation index value of private financing risk in Yantai can be obtained by the following formula.

According to the above monitoring model, we can calculate the value of *RRI*, *SRI*, *CRI* every month during the monitoring sample period and the comprehensive evaluation value *RI* of the model. Therefore, by categorizing the monthly data of the monitoring samples, the corresponding averages and standard deviations of the three types of indicators can be calculated and expressed by $\overset{-}{R}\overset{-}{R}Ī$, *SDRRI*;$\overset{-}{S}\overset{-}{R}Ī$, *SDSRI*;$\overset{-}{C}\overset{-}{R}Ī$, *SDCRI*, respectively; the comprehensive evaluation value and its standard deviation are expressed by$\overset{-}{R}Ī$, *SDRI*. According to the degree of change in the final evaluation value, we divided the risk interval into the following four intervals: the lower risk interval: $RI\leq \overset{-}{R}Ī$; the higher risk interval: $RI<\overset{-}{R}Ī\leq \overset{-}{R}Ī+SDRI$; the high risk interval: $RI+SDRI<RI\leq \overset{-}{R}Ī+2\times SDRI$; the extremely high risk interval: $RI>\overset{-}{R}Ī+2\times SDRI$.

## Experiments

According to the current development situation, it is very realistic and necessary to build a complete and effective private financing risk early warning system. The construction of the private financing early warning model can not only maintain the stable development and improvement of the private financing market, but also monitor the health of the market's survival, detect the abnormal fluctuations of market risk-related factors in time, and provide a reference for making targeted decisions. At the same time, a scientific and effective early warning system can regulate the normal operation of the economy in a long-term sense and provide more appropriate and scientific services for market economic entities, thereby promoting the long-term standardized and healthy development of the private financing market.

### Variable Selection and Data Processing

The structure of each index layer in the whole system of this paper is interlinked. The data fluctuation of the data application layer originates from the data acquisition layer and affects the index change of the index analysis layer, and the changes of the three factors of the index analysis layer determine the Finally, the risk changes in the private financing and lending market, so combined with the concept of information economics “data implicit information, data fluctuations reflect changes in risk”, we can find the reasons for the changes in the risk warning value according to the changes in the comprehensive evaluation value, and then combine The variability of the relevant indicators of the data application layer is to find the most essential reason that affects this factor. This is conducive to the targeted implementation of relevant coordination measures and regulation of them, so as to achieve the purpose of effective risk monitoring.

This article selects monthly data on the comprehensive interest rate of Yantai Private Financing from January, March, June, 1 year and more than 1 year. The sample period is from January 2013 to March 2018. At the same time, the corresponding sample period of Yantai financial institutions' RMB loan balance year-on-year value, *CPI* year-on-year index, and business activity index *ob*_*t*_ in *PMI* are selected as macroeconomic variables. The Bayesian *VAR* model is jointly constructed with the Yantai private lending rate, and its relationship with macroeconomics is studied. Role relationship, in-depth analysis of its information value.

Because in normal research, time series data may have a“pseudo-regression” phenomenon, this paper performs a stationary test on the model index variable series to ensure the validity and robustness of the model estimation results. Table 1 shows the unit root test results of macroeconomic variable indicators:

**Table 1:** Stationary test results of a series of macroeconomic variables.

**Variable**	**ADF inspection**	**Prob**.	**Critical point 1%**	**Critical point 5%**	**Critical point 10%**
CTB	−1.4156	0.569	−3.542097	−2.910019	−2.592645
DFCTB	−15.39	0	−3.542097	−2.910019	−2.592645
Amount	−2.03	0.2736	−3.540198	−2.909206	−2.592215
df Amount	−7.0956	0	−3.542097	−2.910019	−2.592645
Ob	−6.3781	0	−3.540198	−2.909206	−2.592215

DFCTB and dfAmount represent the first order difference values of CTB and Amount, respectively

Based on Table 1, both the CTB and Amount original sequences are non-stationary sequences, and the sequences after the first-order difference are stationary. Ob time series is stable. In the following *VAR* model, first-order difference CTB data and Amount data are used.

## Discussion

### Yantai Private Financing Risk Index Model

**Figure 1:**
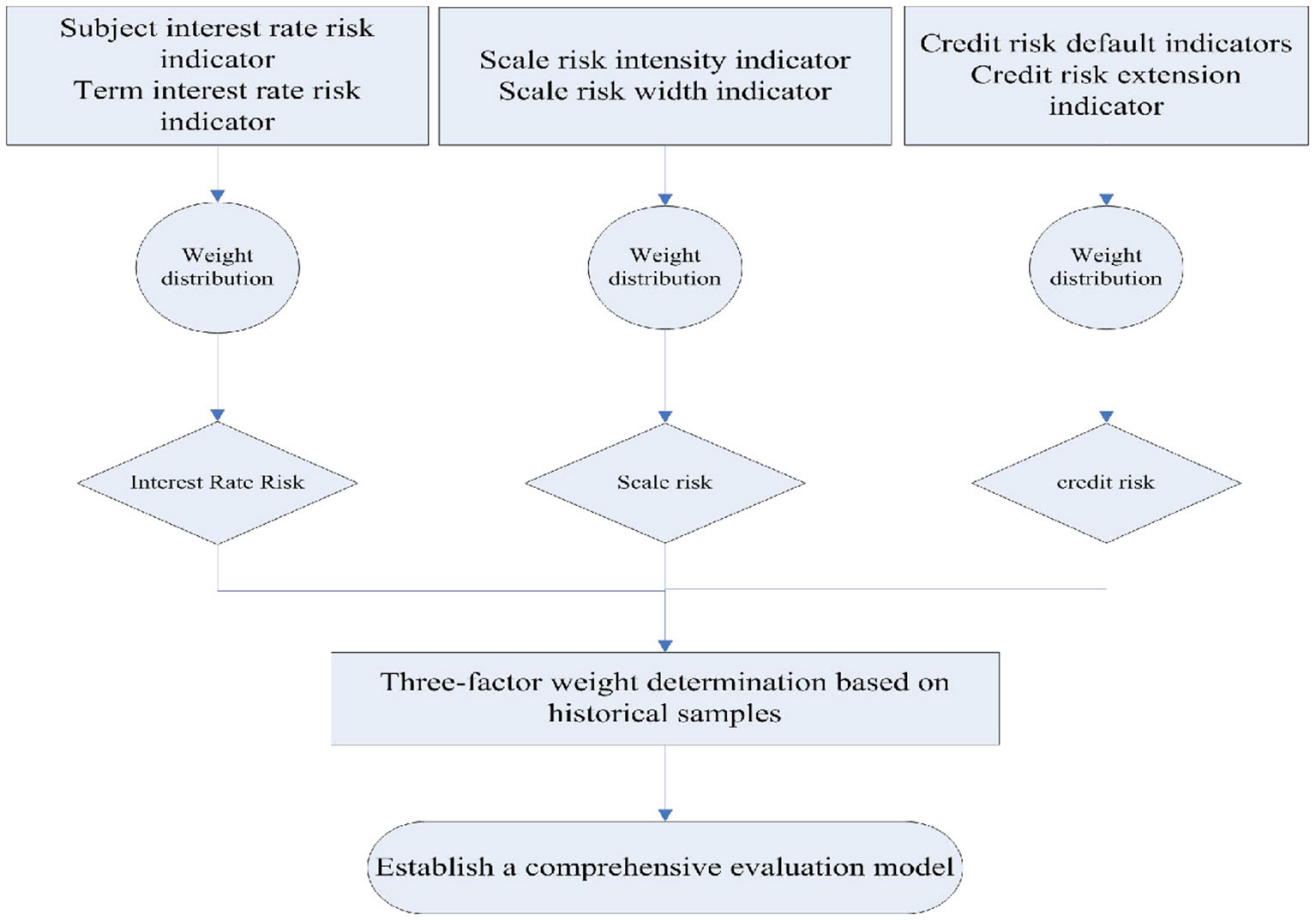
Schematic diagram of the process of establishing a private lending risk model.

The design of the monitoring model is the core of the entire monitoring system, and the results directly affect the correctness and effectiveness of the model monitoring. By referring to relevant annual statistical data (including monitoring data sample data and historical wind disaster sample data), the risks are reflected in the form of quantitative data. The entire model setting process is shown in Figure 1.

### Income of Residents in Yantai

**Figure 2:**
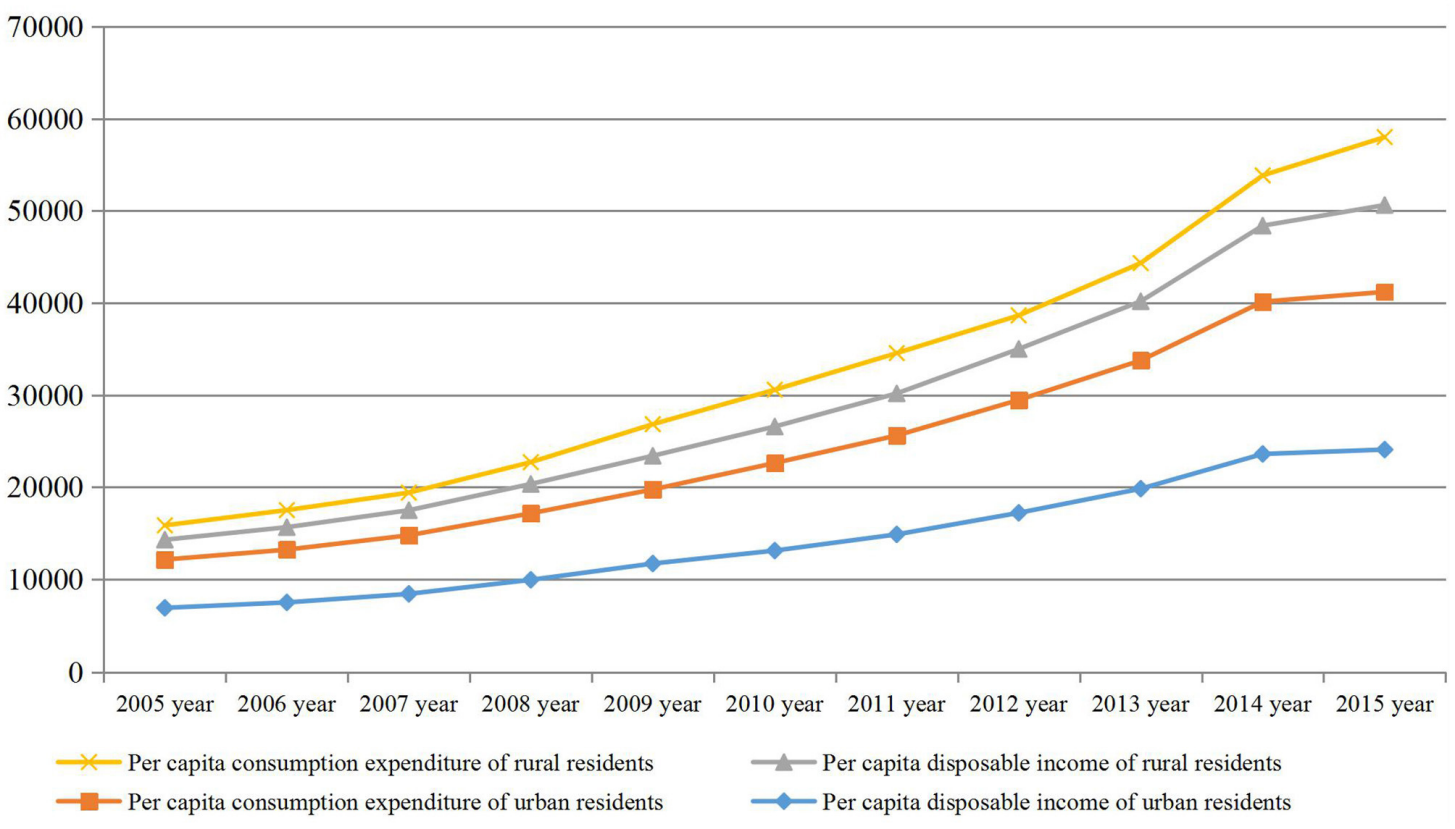
Per capita disposable income and consumption of Wenzhou residents.

According to the statistics of residents' income in Yantai, we can find that the income and savings balance of residents in Yantai have been increasing steadily year by year. The per capita disposable income of urban residents increased by 8.4% annually, and per capita consumption expenditure increased by 8.3%. The average annual net income of farmers increased by 7.8%, and the average annual increase in farmers' consumption expenditure was 8.1%. At the end of the year, the average savings balance of urban and rural residents in the city increased by 9.3%. The year-end savings balance of urban and rural residents in Yantai City was only 12.606 billion yuan in 2005, and increased to 76.483 billion yuan in 2015, an increase of 506.71%. The savings balance between urban and rural residents directly reflects the scale of urban and rural residents' idle funds, and indirectly reflects the scale of capital flows for private financing. Compare income and expenditure, as shown in Figure 2.

### Financial Situation of Yantai

**Figure 3:**
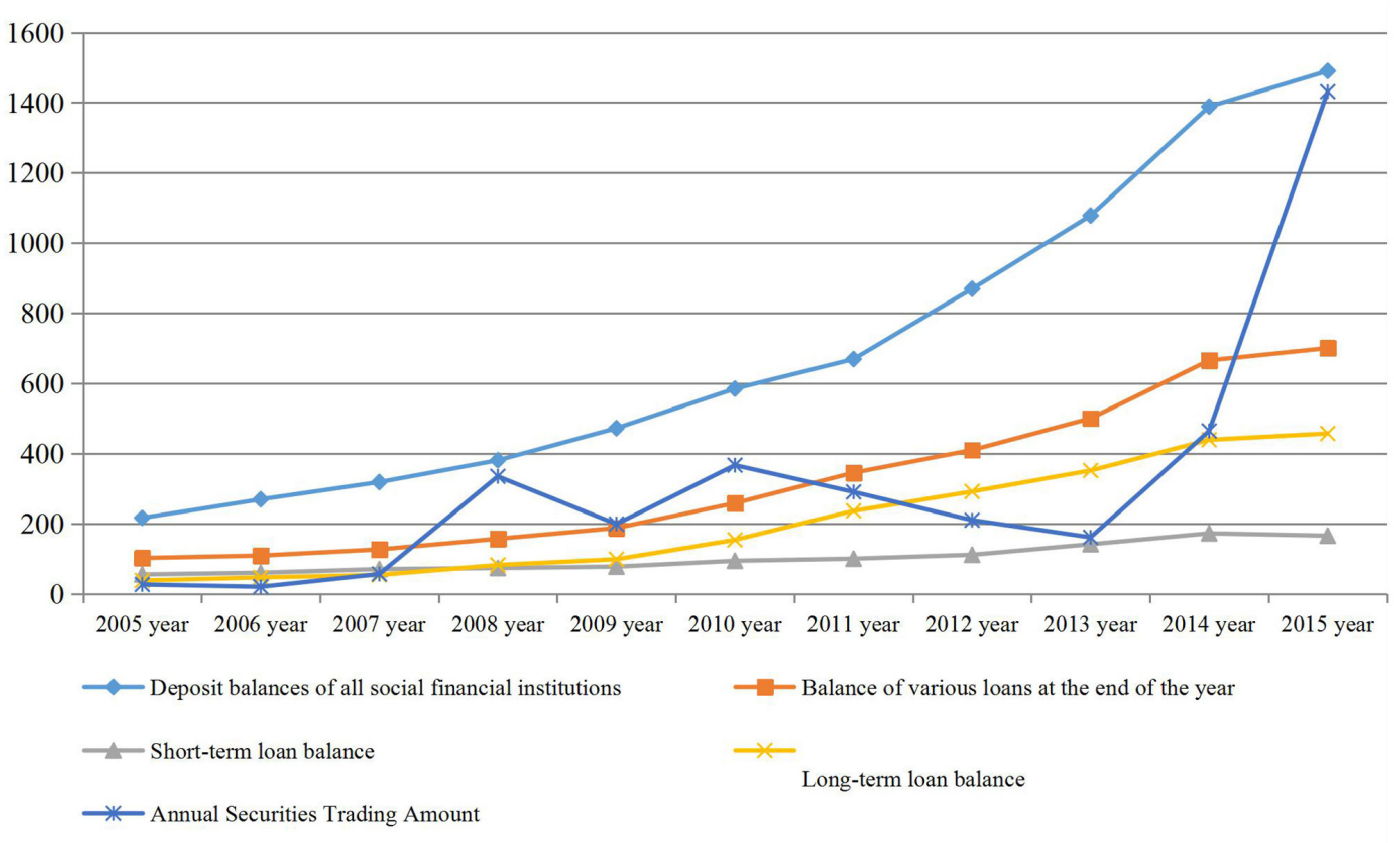
Financial situation in recent years (Unit: 100 million yuan).

With the stable development of Yantai's overall economy, the income of urban and rural residents has gradually increased, and the balance of various deposits in urban financial institutions has grown rapidly. The balance of various deposits of financial institutions in the city increased from 21.612 billion yuan in 2004 to 149.006 billion yuan in 2015, an increase of 589.7%. The balance of various loans at the end of the year increased from 10.181 billion yuan in 2004 to 70.041 billion yuan in 2015, an increase of 58.969%. Among them, the long-term loan balance increased by 11.09 times, while the short-term loan balance increased from 5.496 billion yuan in 2004 to 16.535 billion yuan in 2015, an average annual growth of 10%. The year-to-year increase in securities trading volume has also been rapid, but it is subject to large fluctuations due to the influence of the domestic and international financial environment. An analysis of Yantai's financial situation in recent years is shown in Figure 3.

### Financing Status of SMEs

**Figure 4:**
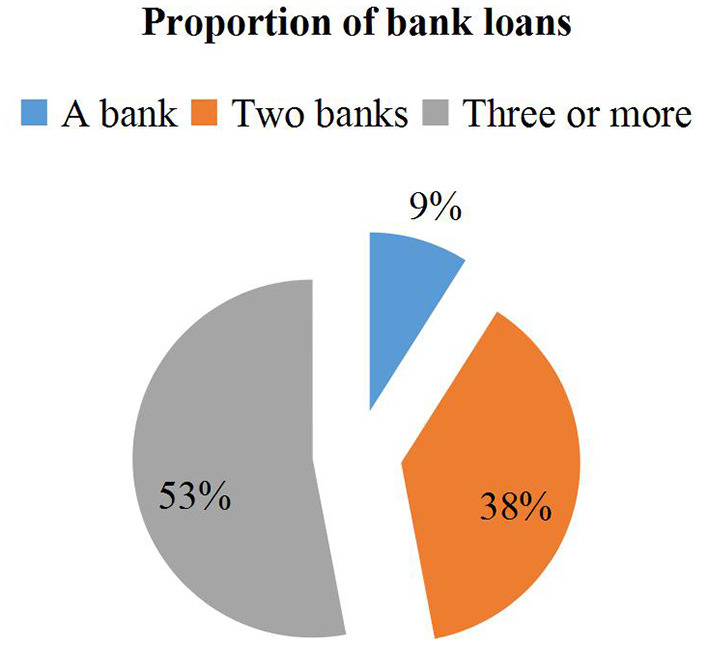
Proportion of bank loans of SMEs.

This article investigates the financing problems of 324 small and medium-sized enterprises (SMEs), and explores the reasons for the closing of SMEs. It is found that the difficulty of financing is the root cause of closing. In the survey, it was learned that 2019 is a year of “closed, running” in Yantai SMEs. After the survey, the country adjusted the banking industry in that year, especially tightened the loan quota. Banks will tend to have good qualifications during the lending process. Large enterprises, resulting in SMEs unable to obtain capital inflows to maintain business operations. In the survey, 98 companies found that financing of SMEs was difficult. Due to information asymmetry, tightening of national bank policies, and mortgage guarantees, well-qualified companies gave up bank financing, but there was adverse selection, and 75 companies were in austerity, Throw in options for high-cost bank financing. For enterprises, only sufficient funds can maintain the normal operation of the enterprise, especially for small and medium-sized enterprises. When this article surveyed 312 companies, it was found that each company has bank lending, and each company is not only affected by the bank of the financier. The sum of the different bank loans of the company is shown in Figure 4.

### Analysis of Private Financing Risk Index Based on Yantai Index

For the indicators involved in the risk early warning, the objective adjustment coefficient analysis is based on the specific time period of the regional private financing market risks, and analyzes the changes of the indicators in different times. First, the risk samples are divided into equal time zones, and the values of the indicators in each period of time are calculated according to the above formula. Then, by comparing different risk samples, the similarity of the data changes before the occurrence of regional risks is comprehensively analyzed, and then the judgment is made. The intensity of the impact of index changes on risk in different time periods is used as an objective adjustment coefficient for potential state risks of different indicators.

The structure of each index layer in the whole system is closely linked. Therefore, according to the influencing factors of the change in the final indicator value, the most fundamental reason for the change in the value of the risk early warning can be found layer by layer, providing a theoretical basis for the supervision of the private financing market.

#### Case Analysis of Yantai's Private Financing Interest Rate Risk Index

In the private financial risk monitoring model, the Yantai index can be divided into six types of interest rate indexes according to the different borrowers: private loan service center interest rate index, small loan company interest rate index, and private loan center interest rate index. Private capital management companies, social direct loan interest rate indexes, rural mutual aid interest rate indexes, and interest rate indexes of other market entities. The standard deviation of the interest rate risk index of each loan entity in the table represents the change in the value of the interest rate index, and it reflects the possibility of potential risks in the loan market. Based on the statistical results in the table, the average interest rate of various types of borrowers can be used to determine whether the interest rate of each month in the sample period is within a reasonable range. If the interest rate value is not within a reasonable range, you can judge that the monthly interest rate is within the above-mentioned risk range. The following sample data is mainly from the release of the Yantai Index. The sample covers data for the 29 months from December 2015 to April 2018. Through the classification and statistics of the source data of the interest rate index, the specific calculation results are summarized in Table 2.

**Table 2:** Interest rates and risks of different lending topics.

**Six types of subject names**	**Sample interest rate mean**	**Standard deviation of subject's interest rate risk index**	**Sample ownership**
Small loan companies	17.74	0.563	0.644
Private capital management company	16.85	0.954	0.006
Social direct lending	16.12	1.243	0.264
Private lending service center	15.96	0.875	0.629
Rural mutual aid	12.09	1.274	0.011
Guarantees, pawns and other markets	26.38	1.182	0.010

And Yantai index can be divided into five types of interest rate index types according to the different loan terms: January period, March period, June period, 1 year period, and 1 year or more interest rate index. Now based on the data of the Yantai Index since its release, the article analyzes the financial risks in the Yantai region through the above financial risk monitoring model. The data basis is the interest rate level and fluctuation of the Yantai Index. The sample coverage period of the interest rate index data of different periods is from January 2013 to May 2018. Calculate the basic data of various interest rate indicators, and then obtain the average interest rate, standard deviation and weight ratio of different loan objects in all samples. The table below shows the results of a comprehensive analysis of interest rates for each subject. The data in the table can be used as a basis for assessing the volatility of the main interest rate risk index, and can also provide a judgment standard for the final risk fluctuation. Considering that the rural mutual aid association is a new topic, the interest rate risk sampling period for this topic is from December 2015 to April 2018. Similarly, the relevant data of the Yantai Index is also classified by period and the relevant statistical results are displayed. In Table 3:

**Table 3:** Interest rates and risks for different borrowing periods.

**The term**	**Term interest rate average**	**Term interest rate risk index standard deviation**	**Weight**
One month	20.35	1.532	0.092
March	18.86	1.256	0.217
June period	17.25	1.155	0.454
One year	15.96	1.107	0.226
More than a year	15.34	1.135	0.010

## Conclusions

Private financing is the most basic, extensive and active financing method. The research on risk monitoring and early warning in the private financing market has always been a hot spot of concern. Effective monitoring of private financial risks will help us to monitor financial market fluctuations. It is particularly important to promote the healthy development of the economy by understanding the situation and discovering the potential risks of the capital market in time, so as to achieve the purpose of preventing the private financial crisis.

To ensure the effectiveness of private financing monitoring and early warning, not only a reasonable system is needed, but also the data support of various financing entities, in order to better grasp the market's regulatory power over financing behavior, which requires diversified samples and reliable collection. channels and the increasingly accumulated data support. With the development of China's economy, the economy of small and medium-sized enterprises has occupied a large proportion of our country's economy. The development of new enterprises has been accompanied by the problem of financing. Coupled with the small size of SMEs, the financial organization structure and low transparency of operations, it is difficult to obtain financing from banks and other formal enterprises, which has led to the generation of private financing. Small and medium-sized enterprises have been increasingly developed, and private finance has become increasingly active in recent years. SMEs have become part of our economy, and private financing can make up for the lack of financing for SMEs. Yantai is not only a gathering place for small and medium-sized enterprises, but also a city with large capital requirements. Many small and medium-sized enterprises in Yantai rely on idle funds to obtain funds and maintain the normal operation of enterprises. Private financing has largely made up for the lack of financing of SMEs, promoted the development of Yantai SMEs, and effectively eased the financing difficulties of SMEs. The emergence of new financing channels means the emergence of financing risks.

This article starts with the Yantai region, diagnoses its risks, analyzes its internal and external risk factors, and proposes corresponding countermeasures to prevent private financing risks. Due to their irrational capital structure and chaotic management, SMEs have difficulty financing. In order to effectively prevent and control risks, enterprises should start to improve their own top-down, including corporate culture, financial management, business philosophy, etc. ;enterprises should strengthen the level of management personnel, achieve division of responsibilities, establish and improve internal control procedures, Scientific management. Reducing the lack of self-management can greatly improve the risk monitoring level of the enterprise, increase the credibility of the enterprise, and increase the possibility of obtaining loans from banks and other institutions, thereby reducing financing costs. At the same time, China should improve the legal system for private financing and establish risk early warning. The system can react immediately when it senses the risk, avoiding big mistakes and difficult to recover. Regulating the interest rate of the private financing market, while reducing its own risk, enterprises can obtain funds within a reasonable range of interest rates to prevent reliance on High private interest rates have resulted in the final profit rolling and unable to bear its financing costs. And in terms of financing terms, interest rates will decrease as the terms increase. The 1-month period, three-month period, June period, 1-year period, and 1-year periods are analyzed. The 1-month risk is compared with the other four. The term is the largest, and the 1-year term has the smallest risks compared to the other four. In terms of financing term, choosing 1 year period is the best, and choosing 1 month period is the worst risk. In addition, combining the model with regional markets is also the key to a more accurate model. Different places have different characteristics, and the average interest rate level, financing activity and methods are different. Therefore, in practical applications, it is necessary to skillfully use the established basic model to improve the work in combination with the characteristics of local financing. This requires not only a deeper understanding of various factors in the market, but also corresponding improvement measures according to the market's response, so that the monitoring and early warning model can keep pace with the times, increase its market adaptability, but also make it more sensitive. By carefully analyzing the relationship between the monitored financing market and various related influencing factors, and gradually adjusting, increasing, and refining the factors used in the basic model according to market demand, the early warning model can be continuously improved to make it more suitable for local financial characteristics, adapt to the actual private financing market environment being monitored.

## Data Availability Statement

The original contributions presented in the study are included in the article/supplementary material, further inquiries can be directed to the corresponding author.

## Author Contributions

JZ: editing data curation and supervision. BL: writing- original draft preparation. All authors contributed to the article and approved the submitted version.

## Conflict of Interest

The authors declare that the research was conducted in the absence of any commercial or financial relationships that could be construed as a potential conflict of interest.

## Publisher's Note

All claims expressed in this article are solely those of the authors and do not necessarily represent those of their affiliated organizations, or those of the publisher, the editors and the reviewers. Any product that may be evaluated in this article, or claim that may be made by its manufacturer, is not guaranteed or endorsed by the publisher.
